# Supplementary material for: Starvation promotes concerted modulation of appetitive olfactory behavior via parallel neuromodulatory circuits
Source: eLife. 2015 Jul 24;4:e08298. doi: 10.7554/eLife.08298 (PMC4531282; doi:10.7554/eLife.08298)
Supplement: Figure 2—source data 1. — DOI: http://dx.doi.org/10.7554/eLife.08298.007 [file elife08298s001.docx]

Figure 2—source data 1

| **Glomerulus** | **Vinegar**  **(% SV)** | **Fed** | **Starved** | **Fed**  **sNPFR-RNAi** | **Starved**  **sNPFR-RNAi** | **Fed**  **DTKR-RNAi** | **Starved**  **DTKR-RNAi** |
| --- | --- | --- | --- | --- | --- | --- | --- |
| **DM1** | 0.2 | 84±3 | 112±7 | 81±3 | 82±5 | ND | ND |
|  | 0.6 | 99±4 | 127±7 | 98±5 | 102±3 | ND | ND |
|  | 1.8 | 117±6 | 133±3 | 120±7 | 119±5 | ND | ND |
|  | 5 | 119±7 | 127±4 | 123±5 | 122±9 | ND | ND |
|  | 10 | 121±3 | 125±6 | 120±4 | 118±5 | ND | ND |
|  | 20 | 118±2 | 122±5 | 111±5 | 116±5 | ND | ND |
|  | 40 | 117±2 | 119±5 | 110±4 | 110±5 | ND | ND |
|  | 80 | 104±9 | 108±10 | 102±3 | 109±3 | 122±12 | 111±15 |
|  |  |  |  |  |  |  |  |
| **DM4** | 0.05 | 18±6 | 36±5 | 12±6 | 7±2 | ND | ND |
|  | 0.1 | 27±7 | 48±6 | 21±7 | 19±3 | ND | ND |
|  | 0.2 | 38±11 | 60±8 | 42±15 | 31±5 | ND | ND |
|  | 0.4 | 45±9 | 71±8 | 56±17 | 38±5 | ND | ND |
|  | 0.8 | 48±6 | 78±7 | 59±12 | 48±5 | ND | ND |
|  | 80 | 80±8 | 87±11 | ND | ND | 98±11 | 93±13 |
|  |  |  |  |  |  |  |  |
| **DM2** | 0.05 | 32±6 | 50±5 | 13±6 | 15±3 | ND | ND |
|  | 0.1 | 48±7 | 70±6 | 20±7 | 29±6 | ND | ND |
|  | 0.2 | 59±6 | 84±7 | 33±8 | 39±7 | ND | ND |
|  | 0.4 | 69±7 | 93±8 | 46±8 | 54±7 | ND | ND |
|  | 0.8 | 73±6 | 95±8 | 50±7 | 53±6 | ND | ND |
|  | 80 | 113±6 | 118±5 | ND | ND | 114±7 | 93±6 |
|  |  |  |  |  |  |  |  |
| **VM2** | 0.05 | 33±9 | 5±3 | 29±8 | 28±9 | ND | ND |
|  | 0.1 | 51±10 | 15±5 | 55±13 | 42±10 | ND | ND |
|  | 0.2 | 70±10 | 37±8 | 67±13 | 55±11 | ND | ND |
|  | 0.4 | 76±9 | 56±10 | 73±13 | 65±10 | ND | ND |
|  | 0.8 | 82±8 | 66±11 | 78±9 | 73±9 | ND | ND |
|  | 80 | 95±3 | 97±7 | ND | ND | 116±6 | 120±6 |
|  |  |  |  |  |  |  |  |
| **VA3** | 0.05 | 0 | 2±1 | 0 | 0 | ND | ND |
|  | 0.1 | 4±2 | 7±3 | 2±1 | 3±1 | ND | ND |
|  | 0.2 | 11±4 | 19±5 | 8±2 | 10±3 | ND | ND |
|  | 0.4 | 32±4 | 38±7 | 23±5 | 21±5 | ND | ND |
|  | 0.8 | 49±5 | 49±7 | 42±6 | 37±7 | ND | ND |
|  | 80 | 42±26 | 58±19 | ND | ND | 53±33 | 79±19 |
|  |  |  |  |  |  |  |  |
| **DP1m** | 0.8 | 0 | 0 | 0 | 0 | ND | ND |
|  | 80 | 68±7 | 70±12 | ND | ND | 55±15 | 73±15 |
|  |  |  |  |  |  |  |  |
| **DM3** | 0.8 | 0 | 0 | 0 | 0 | ND | ND |
|  | 80 | 79±3 | 71±6 | ND | ND | 78±4 | 73±7 |
|  |  |  |  |  |  |  |  |
| **VA2** | 0.8 | 0 | 0 | 0 | 0 | ND | ND |
|  | 80 | 94±8 | 72±15 | ND | ND | 109±9 | 91±9 |
|  |  |  |  |  |  |  |  |
| **DM5** | 5 | 0 | 0 | ND | ND | 4±4 | 0 |
|  | 10 | 8±5 | 0 | ND | ND | 20±9 | 5±4 |
|  | 20 | 23±6. | 2±1 | ND | ND | 24±8 | 19±5 |
|  | 40 | 25±4 | 5±2 | ND | ND | 32±7 | 29±3 |
|  | 80 | 57±9 | 26±5 | ND | ND | 52±12 | 53±5 |

Values are peak ΔF/F (mean±sem) of PN dendritic calcium activity. Values in shaded areas are peak ΔF/F (mean±sem) of ORN axonal calcium activity from a previous paper (Figure 4C in Root et al., 2011). ND, not determined.
